# Charing-Cross Hospital

**Published:** 1852-11

**Authors:** 


CHARING-CROSS HOSPITAL.—*Consulting Physician.* Dr. Shearman, *Physicians*, Drs. Golding and Chowne. *Assistant Physician*, Dr. Rowland. *Surgeons*, Messrs. Hancock and Avery.

MEDICAL AND SURGICAL SCHOOL. *Winter Session.*—Chemistry, Mr. Lewis. Descriptive and Surgical Anatomy, Dr. E. Smith. General Anatomy and Physiology, Mr. Canton. Principles and Practice of Medicine, Drs. Chowne and Rowland. Principles and Practice of Surgery, Mr. Hancock. *Summer Session.*—Practical Chemistry, Mr Lewis. Materia Medica and Therapeutics, Drs. Steggall and Willshire. Principles and Practice of Midwifery, and the Medical Treatment of Women and Children, Dr. Chowne, and Mr. Hird. Medical Jurisprudence, Dr. Birkett and Mr. Hird.

